# From the Cochrane Library: Interventions for Ulceration and Other Skin Changes Caused by Nerve Damage in Leprosy

**DOI:** 10.2196/47148

**Published:** 2023-08-23

**Authors:** Lachlan Anderson, Madeline Adelman, Liv Merete Reinar, Robert P Dellavalle

**Affiliations:** 1 Boston College Chestnut Hill, CA United States; 2 Department of Dermatology University of Colorado Anschutz Medical Campus Aurora, CO United States; 3 Department of Global Health Norwegian Institute of Public Health Oslo Norway; 4 Division of Health Services Norwegian Institute of Public Health Oslo Norway; 5 Dermatology Service Rocky Mountain Regional Veterans Affairs Medical Center Aurora, CO United States

**Keywords:** leprosy, ulcers, zinc tape, nerve damage, ulceration, skin, dermatology, systematic review

## Introduction

Leprosy (Hansen disease) is a chronic and infectious bacterial disease that was responsible for 127,558 new cases worldwide in 2020 [1]. The disease is most prevalent in developing countries, particularly in India, Indonesia, Brazil, Nigeria, and Bangladesh [2]. The pathogenic bacteria responsible for the disease, *Mycobacterium leprae*, infects peripheral nerves, causing nerve damage and sensory loss in up to 30% of patients [2]. This sensory loss can lead to debilitating neuropathic ulcers that can be found on any part of the body, although they are most commonly found on the hands and feet. There are a variety of treatment interventions available [3]. Given the persistence of leprosy as a global disease, physicians must be aware of the most effective treatments for ulcers to prevent further growth and additional infections. A 2019 Cochrane review by Reinar et al [4], “Interventions for ulceration and other skin changes caused by nerve damage in leprosy,” provides a comprehensive review of evidence regarding the effectiveness of various treatments for leprosy-caused skin damage.

## Methods

The review [4] extracted data across 14 studies and 854 patients to evaluate different therapeutic interventions and the associated healing time for leprosy-related ulcers. Of these studies, 13 assessed treatment options for existing ulcers and 2 evaluated the prevention of new ulcers. The studies investigating treatment for existing ulcers assessed interventions including laser therapy, light-emitting diode (LED), zinc tape, intralesional pentoxifylline, pulsed magnetic fields, wax therapy, ketanserin, human amniotic membrane gel, phenytoin, plaster shoes, and protective footwear. The interventions were compared in terms of mean reduction in the ulcer area and the number of ulcers healed over time, with healing time ranging from 2 weeks to 4 months.

## Results

Of the 14 studies evaluated, 3 analyzed the effectiveness of zinc tape compared to other interventions and found that zinc tape resulted in a shorter healing time. However, Reinar et al [4] cautioned that the studies may be affected by bias and wide CIs. Two additional studies compared phenytoin to saline dressing and found that phenytoin resulted in a more significant mean percentage reduction in ulcer area and volume after 4 weeks. Another study examined preventative care by comparing canvas shoes with polyvinyl chloride boots and found there was no significant difference between the two interventions, as none of the 72 participants developed new ulcers over a 1-year period.

The authors note that it is challenging to draw firm conclusions from the results of these studies because most of the studies included had a high and unclear risk of bias. These biases included, but are not limited to, selection, performance, detection, and attrition bias. To improve the quality of future research, larger sample sizes and increased patient follow-up are needed.

Since the publication of this review, there have been several studies exploring further treatments that should be taken into consideration. For example, one study compared the use of topical epigallocatechin gallate (EGCG) 1% to topical antibiotic gauze and found EGCG 1% to be a more effective treatment [5]. Another study looked at the efficacy of a topical insulin solution (10 units) compared to topical normal saline and found the topical insulin to be more effective [6].

## Discussion

The *Journal of the American Academy of Dermatology* does not provide specific recommendations for treating leprosy-related ulcers, emphasizing the importance of early detection and treatment of the disease with oral medications [7]. Preventative measures for ulcers, such as protective footwear, and interventions, including zinc and phenytoin, should be further investigated in areas with high disease prevalence to determine the most effective treatments for patients with advanced stages of the disease [3].

## Abbreviations

EGCG: epigallocatechin gallate
LED: light-emitting diode
